# Hospital and Institutional News

**Published:** 1911-11-18

**Authors:** 


### HOSPITAL AND INSTITUTIONAL NEWS.

### THE SCOTTISH HOSPITALS AND THE BILL.

Elsewhere in this issue we publish a report of an important meeting that has been held in Glasgow to consider how best to secure adequate provision for institutional treatment under the proposed Insurance Bill. Following the policy laid down by the British Hospitals Association, two resolutions were passed unanimously, the first urging the necessity of institutional treatment and the second that such treatment could not be provided if the working men's and employers' subscriptions be withdrawn, as the Bill threatens to withdraw them. We publish the text of the resolutions, and a list of those present, which includes fifty representatives from some twenty hospitals in Edinburgh and Glasgow. The Manchester meeting of the British Hospitals Association has produced many good results, and we hope great vigour will be displayed in other centres besides Glasgow.

### THE ITALIAN RED CROSS AT TRIPOLI.

In view of the hostilities in Tripoli it is interesting to learn from the Italian newspapers how their Red Cross Association is at work. The Red Cross, we learn, has fourteen hospitals in all in Tripoli and Cyrenaica besides the hospital ship *Memphis*. Furthermore, there is at Tripoli an amply furnished magazine with every kind of sanitary material, and medicines, linen, comforts. Ninety-eight of the Association's officers and 605 soldiers have been mobilised and are now in readiness in Africa. The Association has to provide for all expenses, as the Government only transfers to the premises of the Red Cross the rations of provisions appointed for the soldiery. This means of course that the daily outlay tells heavily on the Red Cross's revenue, and once more raises the difficulties of combining voluntary work with governmental control and regulation. At the time of the South African War we were faced with similar difficulties, and it is instructive to watch how the same problems are being solved in the present war. The difficulty is to get news from reliable sources of the true state of affairs.

### LONDON'S NEW MEDICAL OFFICER.

At Tuesday's meeting of the London County Council, Mr. Edward White presiding, Dr. William Heaton Hamer was unanimously appointed as from January next to be Medical Officer of Health and School Medical Officer for the county of London at a commencing salary of £1,250 a year. Mr. H. J. Greenwood, Chairman of the General Purposes Committee, said that though the number of applications, thirty-three, might be considered small, the average of experience and attainment was a high one. Dr. Hamer, whose name we gave last week as one of the selected candidates, has served the Council for nearly twenty years, having gained experience in every branch of the work. At the same meeting the Council approved the appointment of Mr. Arthur Herbert Spicer for a period of one year as Deputy Medical Officer.

### DR. NEWSHOLME ON VITAL STATISTICS AND OFFICERS OF HEALTH.

The annual report of the Chief Medical Officer of the Local Government Board is always full of interest, and Dr. Newsholme this year has some interesting remarks on the improving control of preventible diseases. He draws attention to the declining prevalence of enteric since 1869, and to the special sources of contamination which cause its present occurrence. Tuberculosis, of course, still remains the worst enemy of the sanitary reformer. He shows also that the deaths due to the accidents of child-birth are almost equal to those caused by accidents to adult males. The annual re-appointment of Medical Officers of Health will now cease owing to the new regulations, under which an officer of health appointed for a definite time will continue at its expiration without re-election, but subject to three months' notice. It is hoped that these regulations not only will improve administration, but will free medical officers from the harassing fear that they may not be re-elected.

### THE NEW SECRETARY OF ST. PAUL'S HOSPITAL

We understand that the Secretaryship of St. Paul's Hospital, Red Lion Square, Holborn, rendered vacant by the resignation of Mr. George Gadbury, has now been filled by the appointment of Mr. J. H. Tyler. Mr. Tyler has had considerable experience of hospital work, having been secretary's clerk at the London Hospital, secretary of the London Skin Hospital, and secretary-manager of the Royal Hampshire County Hospital. In addition to the foregoing appointments he was the first secretary of the London Newsboys' Club.

### HOSPITALS AND SCHOOL CHILDREN.

In the chapter in his recent report devoted to the action taken by local education authorities in respect of medical treatment, Sir George Newman, medical officer to the Board of Education, refers to the position of hospitals at length; "the added burden of work arising from the more complete discovery of cures needing the hospital's service is too great for the limited hospital accommodation at present existing, and particularly for the honorary staff. . . . In many hospitals the governors and the medical staff have willingly come forward to undertake the new work and have thereby rendered great service to the children. In other cases the hospitals have not seen their way to do this, and in some cases have even closed their doors to school children, with the inevitable result that the authorities have had to consider the establishment of new institutions for the specific purpose of dealing with school children. Contributions to hospitals and infirmaries towards the treatment of school children were made during the code year ending July 31, 1911, by thirty-four authorities, some of which subsidised a number of hospitals within their education area." Reviewing the whole question of hospital treatment for school children in London, Sir George says there can be no doubt that the hospitals fill, and must continue to fill, an important place in any comprehensive scheme for the medical and surgical treatment of school children. But that place should be in the main a subsidiary one. "In so far as hospitals are used, a portion of the building should be set aside for the exclusive use of the children during the time arranged for their treatment, the necessary officers of the local education authority should be intimately associated with the work, the convenience of the children and their parents should be considered, and the doctor who treats should, in so far as he is treating school children, consider himself in organic relationship with the school medical service generally. In short, the method of the school clinic must be reproduced wherever and however treatment of school children is conducted."

### THE NEW OUT-PATIENT DEPARTMENT AT PADDINGTON GREEN.

We publish elsewhere in this issue the plans of the new out-patient department of the Children's Hospital, Paddington Green, which will be opened soon after we go to press by Princess Louise. As our description points out, "the conditions under which the work of alteration had to be done must have been very restricted indeed," owing to the shape of the site and to the necessity for adaptation. Mr. Alan E. Munby, the architect, has had a difficult task, and the new department which is its outcome will probably have a heavy strain of work to cope with, owing to the long closing that has been necessary, and to the inevitable disorganisation, caused by having to send the hospital's patients to St. Mary's, which has its own difficulties to cope with. The restarting of the work will have, we hope, a brilliant send-off, and Mr. Pearce, the secretary, is to be congratulated on the new addition to his hospital.

### THE CONTROL OF LONDON'S CASUAL WARDS.

The latest step in Mr. Burns's policy of centralisation in Poor Law matters has been taken by the Order, issued on Monday last, which transfers the control of London's casual wards from the Boards of Guardians to the managers of the Metropolitan Asylums District. Mr. H. C. Munro, the Secretary of the Local Government Board, points out in a circular letter that the present arrangement whereby each of London's twenty-eight casual wards is under a separate board of guardians, has led to divergence of treatment in regard to the diet supplied, and the work demanded, which has overwhelmed some wards with applications and left others almost empty. The present transfer, Mr. Munro remarks, was contemplated by the Act of 1871. It would appear, by the way, that this transfer is a slap in the face for the promoters of the Minority Report of the recent Poor Law Commission, for it points to the Board's belief that the grouping of areas under one central authority rather than the classification of those to be provided for among different committees is the best administrative method. The Metropolitan Asylums Board will thus add to its work of running fifteen infectious hospitals, four asylums, eight ambulance stations, a training ship, and the care of certain Poor Law children, the task of looking after the casual, tramp, out of work population of London. The present chairman of the Board and the head of this highly centralised system is Mr. Walter Dennis, a St. Marylebone guardian.

### WEST NORFOLK AND KING'S LYNN HOSPITAL.

Our readers will not have forgotten our account of the surprise visit which the Queen paid early this year to the West Norfolk and King's Lynn Hospital. Now that the King Edward Memorial has been completed there, in the form of an extension, it is noteworthy that the Royal Family have been watching this closely as its opening by Lord Leicester practically coincided with donations from their Majesties. When the Queen surprised the hospital some months ago, she made certain comments which have led to the building of the new staircase that is a prominent feature of the Memorial.

### THE PRESIDENCY OF THE COLLEGE OF SURGEONS.

The medical profession and the institutional world will alike receive with regret the news that Sir Henry Butlin has found it necessary to resign the presidency of the Royal College of Surgeons owing to ill-health. The College has had no lack of presidents distinguished for academical eminence, worldly sagacity, and a fitting dignity; but few of its titular heads in recent years have combined these attributes in better proportion than the great surgeon whose services the College is now losing. As a speaker Sir Henry Butlin has fulfilled the requirements of his post admirably; in this direction some of his predecessors, otherwise admirably efficient, have been much less gifted. As a man of affairs, as a genial and winning personality, and as a thoroughly practical and scientific clinician, his success has been not less notable. Sir Henry Butlin's hospital associations are mainly with St. Bartholomew's, where he was surgical registrar in 1872, assistant surgeon in 1881, full surgeon in 1892, and consulting surgeon in 1902 after thirty years' service. It may be hoped that this resignation does not imply any retirement from the large practice which Sir H. Butlin has long enjoyed. The newly elected president, Mr. R. J. Godlee, is almost as well known as his retiring predecessor. Surgeon-in-Ordinary to his Majesty the King, member of the Senate of London University, Surgeon and Professor of Clinical Surgery at University College Hospital, and Consulting Surgeon to the Brompton Hospital for Consumption and Diseases of the Chest, Mr. Godlee has been especially interested in thoracic surgery, to the advancement of which he has himself contributed. The traditions of the College are certainly safe in his hands, and although he has a difficult predecessor to follow, there can be little doubt that he will rise amply to the occasion.

### DENTAL INSPECTION AND ANÆSTHETICS.

The Education Committee of the London County Council has decided, subject to the approval of the

Board of Education, to renew for one year the agreements with the Deptford Children's Health Centre and the St. George's Dispensary, Blackfriars, for the dental treatment of school children. The sub-committee, which reported on the matter, stated that in view of the intending reorganisation of the Council's medical work, it was not proposed to deal with the general question of the dental treatment of children at present, but the two centres were quite suitable for retention as part of a permanent scheme. All the dentists concerned agreed that an anæsthetic was essential in difficult cases of extraction; but no provision has been made for dealing with these cases, and so when necessary at all the centres treatment has been withheld. The Council, however, as already reported in The Hospital, in August sanctioned the employment of an anæsthetist in connection with the treatment at the Poplar Hopital, and it is proposed that this provision shall be extended to all the schools. We hope that this common-sense course will not be delayed indefinitely.

### HOSPITAL ABUSE IN INDIA.

The Report on Sanitary Measures in India for 1909-10 (Cd. 5770), just issued, states that the extent to which hospitals are supported by the inhabitants varies very much in different parts of the country; in Bengal (excluding Calcutta) subscriptions from Indians amounted to £13,560 and covered nearly 20 per cent. of the expenditure; on the other hand Madras only produced less than 1½ per cent., and Bombay less than one. There is no evidence of a decline in popularity of the existing hospitals; on the contrary, the number of patients treated during the year, which was a healthy one, was nearly a million more than in 1908. Every province reports that women and female children are applying for assistance more freely than formerly; but the well-to-do continue to take advantage of the dispensaries as well, and thereby retard the growth of an independent medical profession outside big towns.

### CHRISTMAS FOR THE LONDON CRIPPLES.

Sir William Treloar is early in the field this year with his annual appeal on behalf of the crippled children of London, for whom he has already done so much. Every year his distribution of hampers, provided by the special fund which he has organised, brightens the lives of over thirty thousand of the poor of the Metropolis; every year, too, he entertains, by kind permission of the Corporation, some 1,200 crippled children at the Guildhall. Moreover, his interest in the fund and the conditions that called it into being has been responsible for the founding of the fine Cripples' Home at Alton which may claim to rival the famous Widener Institute at Philadelphia. Sir William, in the letter that accompanies his appeal on behalf of the fund, informs us that he has received a letter from Lieutenant-Colonel Sir William Carrington stating that the King is once more sending an annual subscription to the fund—one of the best testimonials, surely, as every institutional worker knows, to the fact that the appeal is deserving of general public support. It is Sir William's hope (and one which we earnestly trust will be realised) that he may this year be in a position to satisfy every deserving applicant, and the balance in hand is to be devoted to the Cripples Home at Alton. The Mayors of the Metropolitan boroughs are co-operating with him in distributing the hampers on the widest possible basis. All donations, however small, will be gratefully received by, and should be sent to, Sir William Treloar's Little Cripples' Christmas Hamper and Clothing Fund, 6 Ludgate Hill, E.C. We commend this appeal to all our readers in the hope that the eighteenth distribution (for Sir William has carried on his good work for seventeen consecutive years now) will be a record one, such as in Coronation year it ought to be.

### GIFTS TO HOSPITALS BY AERIAL POST.

The Duke of Teck, who has become trustee of the Aerial Post Fund, has received £1,450 from the organising Committee, the majority of which, we learn, is to go to the King Edward VII. Hospital at Windsor. It is interesting to see how closely the hospitals have learnt to associate themselves with every new movement, and it is worth recording that this is, we believe, the first hospital to receive a bequest by means of the Aerial Post.

### THE MEDICAL COUNCIL ELECTION.

By the time these lines appear in print the election of direct representatives of the profession in England will be concluded, for the last day on which votes may be recorded is Wednesday, November 15. It is not so likely that the result will be declared, since the number of votes to be counted will certainly be very large, probably much larger than at previous elections. To the candidates and their records in an institutional sense reference has already been made (The Hospital, November 4, p. 117); there are, however, one or two curious facts which are apparent from a study of the final list as it was submitted to the electors. Thus there is not a single M.D. of London, Edinburgh, Oxford, or Cambridge University among the candidates. Indeed there are only three M.D.s in all; one of these is a Birmingham qualification, and the other two are Durham, the latter apparently of the kind which that University thinks fit to grant to practitioners of some years' standing, no matter what their previous education or qualifications. Then there is not a single Fellow or Member of the Royal College of Physicians, nor a single Fellow of the Royal College of Surgeons. In so far as this may be taken to show that the candidates are all genuine specimens of the general practitioner, it is perhaps not matter for great regret; yet there are many general practitioners who possess one or other of these diplomas. So, too, the mere lack of candidates holding the doctorate of our oldest and best Universities is not necessarily anything to deplore; yet at the same time, taking them all round, the men educated at those Universities are men of slightly wider views and higher culture than those whose qualifications are collegiate only. At any rate, the absence of representatives of those Universities and of the governing bodies of the colleges is somewhat remarkable.

### THE CHRONICLER OF GUY'S

This title not inaptly may be applied to Sir Samuel Wilks, whose death last week to many members of the hospital world was the removal of a trusted personality. It is not as past President of the Royal College of Physicians, or as Physician Extraordinary to Royalty that we would deal with him here. It is the editor of Guy's Hospital Reports and the author of a volume of scientific reminiscences that we wish to emphasise. Few medical men have had their lives so intertwined with that of their hospitals as Sir Samuel Wilks' has been with Guy's, and it was thoroughly characteristic of him that his recent volume of memoirs was essentially only the Guy's Reports seen from another and more intimate side. This type of hospital physician is not unfortunately a growingly numerous one, but the personal influence of this example is eloquent of its power for good. At the funeral Guy's Hospital was represented by Mr. H. Williams, clerk to the governors, and Dr. Frederick Taylor, Sir James Goodhart, Dr. Hale White, Dr. Newton Pitt, and Mr. Symonds, representing the medical and surgical staff.

### THE STANDARD OF APPEAL WRITING.

We all know how difficult it is to make an annual appeal annually attractive, and therefore it gives us no pleasure adversely to criticise even the worst. Nevertheless, in the interest that we have in maintaining as high a standard as possible in this particular form of charity advertising, praise or blame must be given critically, especially to those institutions which make a specialty of appeals. The Royal Hospital for Incurables on Putney Heath has set in the past a commendably high standard. Last year in particular its republication of Charles Dickens' arguments on its behalf deserved and received, from us at any rate, very sincere praise. An advance copy of this year's Christmas appeal lies before us. It is called "Our Pensioners," and strikes a new note, as its title implies, by referring exclusively to the pensioners, and by leaving the in-patients alone. The idea is praiseworthy, as many people are not aware of this branch of the work. The idea is carried out by the carefully crossheaded statements of those who have personal knowledge of some of the Home's beneficiaries. Which, we ask, have been selected? Those, we fear, it must be replied, whose cases make the most crudely sensational reading. Thus the crossheaded titles of these scraps of biography include "Through five operations," "Thirty-two years of pain," and "Shapeless all over"—to give the worst examples. Now, from a charity which on the whole understands the art of appeal writing so well, this sort of thing is not excusable. There is neither style nor imagination about it; and no appeal—witness the previous ones issued under the same auspices—has ever suffered from the possession of these. Does it even pay? We venture to doubt it, subject to one condition: that the same trouble be taken to appeal to the brains and the more cultivated emotions as has been taken here to collect horrors. A high standard of appeal writing is worth fighting for, and worth maintaining. That is why we take this little pamphlet so seriously.

### KING GEORGE AND KING EDWARD'S FUND.

His Majesty has not left for India without approving the appointment of a committee to exercise the functions of the Governors, two of whom, the Duke of Teck and Viscount Iveagh, are to be present at the Coronation Durbar. The Speaker of the House of Commons has concurred in the arrangement by which the Earl of Donoughmore, Lord Chairman of Committees of the House of Lords, and the Rt. Hon. Sir T. Vezey Strong, Bt., K.C.V.O., will act with him as a Committee exercising the powers of the Governors until their return from India.

### THE LATEST EFFORT AT HARTSHILL.

Our readers will be aware that the complete reconstruction of North Staffordshire Infirmary was undertaken as the result of a Report published in The Hospital almost exactly two years ago. Local feeling was strongly aroused, to which we were the convenient buffer, and £33,000 was raised. Two thousand more is needed to complete the architectural rearrangement which Mr. Keith Young has drawn up, and a meeting at the neighbouring township of Leek has been held to start a fund for the purpose. At this meeting Colonel A. H. Heath, this year's president of the hospital, remarked on the ability shown by Mr. Basil Rhodes, the secretary and house governor, and by the medical staff under very difficult circumstances. He also stated his belief that "the visit of Sir Henry Burdett has not done us any harm, because his severe criticism has shown people how very necessary it was that a complete renovation should be undertaken." We wish the Leek Fund a speedy task in raising the additional money.

### AN EXTRAORDINARY PROTEST MEETING.

Ought the Managing Committee of the Royal Hospital for Incurables, Donnybrook, Dublin, to provide additional accommodation in a new pavilion or wing, and in the present hospital grounds, for patients dying of consumption? One would hardly think such a proposition likely to create an uproar, and to cause the holding of a protest meeting so biassed as to listen to nothing but its own outcry. Such, nevertheless, has been the case, and, with Dr. Donnelly, Bishop of Canea, in the chair, the dictum solemnly went forth that the meeting must confine itself to the duty of protesting, in spite of the fact that more than one person had arguments to put forward on the other side. The arguments of the reverend Bishop, and of Mr. J. H. Edge and Mr. James O'Connor, K.C., the proposer and seconder of the protesting resolution, may simply be summed up as fear of infection, and yet, can it be doubted, everyone present at the meeting would be prepared to enjoy a winter at Davos, or a house in the Brompton Road, if they wanted a change and could afford to pay for it. Mr. James Mahoney, in a very capable statement which he handed to the newspapers (as he was ruled out of order at the meeting), presents a most cogent argument, in which he points out, among other things, that the proposed pavilion will be further removed from all neighbouring dwellings than the present female consumptive wing, and that in the whole history, except in one possible instance, no complaint has ever been made of infection arising from the presence of consumptives in a city. These arguments, so far as we are aware, the protesters have been quite unable to impugn.

### A WARNING TO LIVERPOOL CHAPLAINS.

Again the total sum subscribed through the Liverpool churches on Hospital Sunday has declined, and this notwithstanding the fact that 421 instead of 403 churches in 1910 held offertories for the purpose this year. Last year £14,530 was subscribed; this year it is only £13,800, in spite of the fact that a donation of 10 per cent. was offered on any increase in the total collections of the joint funds. It is, we fear, idle to accept the facile excuses that have been offered; that Church people have moved into the country, that the churches have been passing through a critical time, and the like. As long as the churches believe they have a serious work to do, so long will times be critical for them. It must be so with every fighting cause; and, as for the second reason, that population is migratory is no new thing. The appeal for Hospital Sunday does not always ring now with its old enthusiasm from the pulpit, and it will not do so until hospital chaplains again take the lead by inspiring their sermons with the driving force of the voluntary spirit. Again, we repeat, hospital chaplains have duties as imperative outside the wards as those within, and to them the hospitals must look as the pioneers of a resuscitated Hospital Sunday. Hospital boards would be well advised to point out to them their responsibility in this matter, and to hold them, as they hold every other member of the staff, to strict account. Here is a work for the Fund's Committee, for the Rev. E. Walsh and his brother clergy, Mr. J. T. Mitchell and Mr. Linton Smith, to take in hand.

### THE BOX SYSTEM.

Dr. Koplik, of "Koplik spot" fame, writes an article in the *Archives of Pediatrics* on Hospitals for Children, and expresses himself in favour of the box system. This system is based upon the view that all the exanthemata are contagious and that atmospheric infection does not occur. Dr. Koplik expresses himself very strongly upon this point. He states that a study of the "box system" at the Pasteur Hospital "is a convincing argument against the potency of atmospheric infection. What amusing and annoying blunders and injustice have been committed in the name of the atmospheric infection theory, the life-work of every sincere man will show." It is further added: "in America we can see that the times are still with the atmospheric as well as with the contact theory, and while admitting the importance of contact infection the mysterious fetish of atmospheric agencies certainly holds in fetter those whose influence counts for something." When such a deeply rooted belief is held in the fallacy of atmospheric infection advocacy of "the box system" becomes easy. References are made to the system as worked at several hospitals, but more especially at the Pasteur Hospital. In that hospital, apparently, infections occurring within the hospital are rare. It is said that in ten years there were thirty infections in 9,677 patients. Statistics are also given of the death-rate in the various common infectious diseases. Commenting upon these statistics, Dr. Koplik states, "I know of no such favourable statistics or anything like them elsewhere." The "box system," compared with that of isolation wards in ordinary children's hospitals, is largely a matter concerning expense of building and of the nursing staff. As Dr. Koplik says, "it is a spiritual comfort to banish a case of measles to where not a soul can get it," but the proceeding probably does not provide much comfort for the child or for the nurse commissioned to solitary confinement to look after it. On the other hand, nursing under the box system—both contagious and non-contagious cases being attended to by the same nurse—requires great care upon the part of the nurse, but this care may be useful in the training of a nurse for dealing with all cases, whether primarily infectious or not. It may be remarked that at the Pasteur Hospital there is a complete sterilisation daily of eating and other utensils supplied to the patients.

### A USE FOR CRITICISM.

A chance, if familiar, criticism provided Colonel Bruce Vaughan with a text at the monthly meeting of King Edward VII.'s Hospital, Cardiff. It was suggested that the management of the institution was unsound, because continued appeals for support were found necessary. The Colonel, having pointed out that nearly 1,000 patients were waiting for admission and that the new wing was ready for their reception, if the public would subscribe the money for its maintenance, announced that the King Edward Memorial Fund, stimulated by the recent challenge of "A Glamorganshire Owner of Property," had now reached a total of £21,118, leaving £882 to complete the first object of the fund—viz. the liquidation of the debt on the infirmary. It was encouraging, in view of the work before them, to look back for a moment upon what had already been accomplished. Within the past few years they had opened fifty beds, making a total of 196. The accommodation provided by the new wing will now bring the total up to 278 beds. Notwithstanding all the extra outlay involved in the maintenance of these buildings, their increased income from various sources (with the huge debt now almost paid off) would cover the cost of maintenance, at any rate for this year. They were financially sound to-day, but there was yet much to do, and many sacrifices to make. The efforts made at Cardiff are an object-lesson of what the voluntary spirit can do. We well remember announcing the overdraft in these columns, which is why we are glad now to announce its liquidation.

### MAGGOTY PATENT FOOD.

A RECENT inquest on a child aged three months revealed the fact that maggots had been found in the patent food upon which it had been feeding. The child was stated by the medical attendant to have died of enteritis, and the jury returned a verdict of "Death by misadventure due to eating maggoty food." The presence of maggots in patent food must be very unusual. Possibly a beetle the grubs of which feed on cheese had, in the shop from which the packet was bought, found its way into the packet and laid eggs there. But the eggs having been laid and hatched, it seems reasonable to suppose that more moisture than is usually present in dried patent foods would be necessary for the life of the grubs. Although the presence of the grubs may not have been so serious a matter as the jury supposed, the possibility of patent food becoming damp raises the question whether patent foods stored in shops or hospital dispensaries may not contain unsuspected elements of danger. A damp patent food would provide a good culture medium for micro-organisms, and although those micro-organisms would generally probably be harmless, there is always a risk of some dangerous microbe being present. In the case to which reference has been made the acute illness is said to have commenced on the day the food was taken; and the connection of the illness with the food was consequently considered by the medical attendant to have been definite. The time of year in which the fatality occurred suggests that the possibility of coincidence cannot be altogether excluded. Yet so little is known of the nature and life-history of micro-organisms causing epidemic diarrhœa in children that it cannot be stated that the micro-organisms of this disease were not growing in the maggoty patent food.

### THE DUTIES OF A HOSPITAL PHARMACIST.

MR. F. A. HOCKING, B.Sc., Pharmaceutist to the London Hospital, made the other day some interesting remarks on this subject. Twenty years or so ago, he pointed out, the hospital dispenser was not infrequently a glorified porter, devoid of education and training, who worked in a cramped dispensary, ill adapted and indifferently equipped for the work he had to do. In recent years, however, the dispensing department has been re-created. Each hospital of any size has its own collection of formulæ contained in the Hospital Pharmacopœia, and from this work prescribers generally select their prescriptions. Occasionally a preparation can be obtained on the market quite as cheaply as it can be made, and in such a case the advantage of manufacturing over buying becomes a matter for discussion. One of the most important duties of the chief dispenser is connected with the purchase of supplies. At the London Hospital three forms of tender are used; one for drugs embodying some 230 items, another for dressings with over 50 items, and the third for glassware with 50 items. To each tender are attached particulars of the terms and conditions upon which contracts will be accepted. The pharmacist is almost overwhelmed by an avalanche of samples of all kinds and sizes, and for some weeks is almost wholly engaged in tabulating prices, examining and testing, and compiling a report. The work involved at contract time, although laborious, is most interesting. The dispensary of a small hospital is, in the main, a replica of the dispensary of the larger institution, but the duties sometimes include matters unconnected with pharmacy, such as an occasional milk analysis, photography of interesting cases, and possibly, assisting the radiographer. Mr. Hocking's wide survey appeared only to omit reference to a hospital pharmacists' relation to the patients. But it is this which furnishes the humorous and human side of his profession.

### THIS WEEK'S DRUG MARKET.

A SOMEWHAT quieter tone prevails in the drug market, and for the time being no article attracts especial notice. Changes in value have been few. Menthol, the price of which advanced so rapidly a week or two ago, is very quiet, and it is not impossible that before many weeks the article will be procurable at much more reasonable prices; there can be no doubt that the high rates at present ruling have a restrictive effect on the demand. There is no alteration in the price of sugar of milk, but it is thought possible that a slight reduction in value may take place shortly, and under the circumstances it would be as well to postpone purchasing the article for the present, if convenient to do so. A fair demand for cod liver oil exists, and prices are still inclined slightly downwards. Since last week's further advance in the price of santonin there has been no alteration in the position, and another rise in value is hardly considered probable at present. The value of extract of malt is tending upwards in consequence of the moderate barley harvest. Croton oil, a drug which is in much smaller demand nowadays than was the case formerly, is dearer. Oil of cubebs is rather lower in price. The high price of opium is very firmly maintained, and morphine is somewhat dearer; there is no alteration in makers' quotations for codeine, but in view of the position of opium an advance in price is not improbable, notwithstanding the high figure at which the alkaloid is now quoted. Balsam tolu is again dearer. It is still thought probable that an advance in the price of cocaine will take place. Hydrastis canadensis is in rather better supply and the price is a trifle lower.

